# Predictive Analysis of the Mechanical Properties of Biopolymer–Fiber-Reinforced Composite-Stabilized Soil Based on Genetic Algorithm-Optimized Back Propagation Neural Networks

**DOI:** 10.3390/polym17162176

**Published:** 2025-08-08

**Authors:** Guotao Wei, Zhaoping Wang, Xuanhao Cao, Jiuran Wen

**Affiliations:** 1 No. Three Engineering Co., Ltd. of CCCC First Highway Engineering Co., Ltd., Beijing 101100, China; weiguotao@ccccltd.cn (G.W.); wangzhaoping@ccccltd.cn (Z.W.); 2 School of Materials Science and Engineering, Chang’an University, Xi’an 710061, China; caoxh@chd.edu.cn

**Keywords:** soil stabilization, biopolymer, genetic algorithm, prediction model, mechanical properties

## Abstract

The limitations imposed by the inherent complexity of multi-component composition ratios in biological polymer-stabilized soils have hindered rapid and accurate performance prediction. To enhance the predictive accuracy for biopolymer–fiber-stabilized soils, an optimized GA-driven backpropagation (BP) neural network was developed. Three key factors influencing mechanical strength (guar gum (GG), xanthan gum (XG), and polybutylene succinate (PBS)) were identified. The global optimization capability of GA was utilized to construct an integrated GA-BP model, with these factors serving as inputs and 7d compressive strength as the output. Support vector machine (SVM) was also incorporated to provide a benchmark comparison of predictive performance. Validation was performed using 80% of the dataset, with the remaining 20% used for testing. The optimal biopolymer dosage was found to be within the range of 0.5% to 1.0%, and the maximum 7d compressive strength achieved was 466.67 kPa at the 0.5% XG–0.5% GG combination, representing a 273% increase over untreated soil. The GA-BP model demonstrated superior performance in terms of prediction accuracy and stability, as indicated by an R^2^ of 0.887—significantly higher than those of the BP (0.714) and SVM (0.554) models. The mean squared error was substantially reduced to 1413, compared to 2130 and 3113 for BP and SVM, respectively. Although MAPE approached those of the GA-BP, the overall predictive efficacy of SVM was found to be inferior. A reliable and robust methodology for forecasting the mechanical behavior of stabilized soils has thus been provided by this model, supporting advanced applications within geotechnical engineering.

## 1. Introduction

As a distinctive soil type extensively distributed across semi-arid regions worldwide, loess faces a multitude of unique challenges in geotechnical engineering that demand urgent attention. Its large void ratio, high compressibility, and pronounced propensity for collapse under water conditions are attributes that severely limit its direct application in engineering practices due to its untreated, unsaturated, and uncompacted structural characteristics [1,2]. These properties render its framework loose, with weak cohesion between particles, making it highly susceptible to rapid strength loss when exposed to water, which can subsequently lead to subsidence and deformation, thereby threatening the stability of foundations [3,4]. Consequently, the development of efficient and environmentally friendly stabilization techniques aimed at improving its strength and stability has become an urgent and critical issue within the domain of geotechnical engineering.

While cement-based stabilization demonstrates proven efficacy in enhancing soil strength through hydration reactions, its environmental footprint has become increasingly problematic. Each ton of cement production emits approximately 0.8–0.9 tons of CO_2_, contributing to 8% of global anthropogenic carbon emissions [5]. Moreover, the energy-intensive clinker burning process consumes vast quantities of nonrenewable resources. The existence of these issues severely hampers the safety and sustainable development of infrastructure in loess-rich regions, highlighting the urgent need for effective remedial and improvement measures.

In recent years, biopolymers have garnered widespread attention due to their environmental friendliness and sustainability [6]. Biopolymers such as xanthan gum (XG) and guar gum (GG) have garnered significant attention from researchers due to their efficacy in enhancing the soil consolidation capacity [7]. XG is soluble in both cold and hot water, enhancing the viscosity of the matrices it binds with [8]. Additionally, it possesses various desirable attributes, including pH stability, storage stability, compatibility with ionic salts, pseudoplasticity, and strong shear stability even at low concentrations [9]. Like XG, GG is soluble in water. GG is characterized by a higher number of galactose branching points compared to many other biopolymers, making it an excellent stabilizer. The molecular structure of this gum enhances the viscosity of liquids [10,11]. Studies have shown that a 2% incorporation of XG and GG can enhance the liquid limit of clay by 8.0% and increase the shear strength by over 30% compared to untreated soil [12]. The compressive strength of the soil was augmented by over 200% through the stabilization treatment employing β-1,3/1,6-glucan biopolymer, as reported by Chang [13]. Additionally, the water-retaining properties of XG can suppress moisture evaporation, prolonging the stability of soil, while the superior adhesion capabilities of GG show better performance in reducing crack width in loess [14]. These characteristics position bio-based gels as promising alternatives to traditional chemical stabilizers. However, its sole application is prone to insufficient long-term water resistance due to its water solubility. Relevant studies have demonstrated that composite blending with biopolymer formulations can be employed to overcome the performance limitations inherent to individual materials. Research conducted by Jalal et al. has demonstrated that the incorporation of cross-linked biodegradable polybutylene succinate (PBS) and XG composites can significantly enhance the mechanical strength of residual granitic soils, reduce soil erosion in arid regions, and improve soil stability. The UCS of PBS–XG-treated residual granitic soils exhibited a 57% increase after 90 days of curing [15]. Although biopolymer co-blending remains a viable approach for soil stabilization, in-depth investigations into the synergistic reinforcement mechanisms are still requisite.

Furthermore, in the application within loess regions, the exclusive use of bio-based cementing materials for soil stabilization has demonstrated certain inherent limitations. This degradation can adversely affect the stabilization effectiveness. In addition, the capacity of bio-based cementing materials to enhance soil crack resistance is limited, making it difficult to fully suppress crack propagation caused by long-term environmental temperature fluctuations. In response to this issue, fiber reinforcement technology has been introduced into soil improvement practices [16]. Research has indicated that the synergistic effects between fibers and bio-based gels can create a three-dimensional network structure, restricting particle movement and distributing stress, thereby significantly improving the unconfined compressive strength and the soil’s resilience to environmental moisture fluctuations [17]. When XG was combined with enzyme-induced carbonate precipitation to strengthen calcareous silty soil, the addition of fibers resulted in a reduction of strength loss by over 40% under variable moisture conditions [18].

However, the enhancement of the mechanical properties of polymer composite-stabilized soils within multicomponent systems is complicated by the intricate composition ratios of the polymers, rendering the final outcomes inherently difficult to forecast. Traditional approaches heavily rely on empirical formulas, experimental data, and numerical models for prediction. When dealing with variable factors and complex conditions, these methods are frequently challenged by their limited capacity to accurately predict the effects of polymer hybridization on soil strength. Moreover, to avoid the costs and time-consuming nature of traditional laboratory testing procedures, numerous researchers have shifted their focus from routine experimental methods toward mathematical modeling strategies.

In recent years, machine learning algorithms—such as artificial neural networks, deep learning, and support vector machines—have been extensively employed in performance prediction and analysis [19,20]. These algorithms exploit advanced computational capabilities to decipher complex patterns and relationships embedded within large datasets [21,22]. The decision tree-guided artificial neural network pretraining (TANNP) method proposed by Zhang is employed to train a high-precision model for predicting the compressive strength of concrete using a small-scale dataset [23]. The results demonstrate that, compared to the direct application of a neural network, the TANNP method achieves a reduction in the root mean squared error (RMSE) from 5.10 MPa to 3.26 MPa, representing a decrease of approximately 36.1%. Similarly, Mohammed et al. utilized artificial neural networks (ANN), M5P trees, and regression analysis techniques to predict the early-age compressive strength of polymer-modified concrete [24]. The results indicated that the ANN model demonstrated superior predictive performance in estimating the concrete’s compressive strength. Additionally, numerous scholars have adapted BP neural networks for applications such as temperature prediction and behavioral analysis, demonstrating the versatility and superior predictive performance of BP neural networks relative to conventional empirical models [25,26]. However, due to the variability in polymer types and the diversity of their composition ratios, the distribution of original data tends to be broad, making it difficult to construct predictive models based solely on simple linear relationships. General-purpose machine learning algorithms are often unable to effectively capture these complex, nonlinear interactions. Notably, the performance of different machine learning algorithms exhibits selectivity toward various feature types within the datasets. Consequently, the development of models capable of accurately predicting the influence of multicomponent polymer compositions on fiber-reinforced stabilized soils’ compressive strength becomes critically important for the optimization and precise implementation of geotechnical engineering practices.

In view of this, this study systematically investigates the mechanical properties of a biopolymer–fiber-reinforced stabilized soil system, utilizing XG, GG, and PBS as biopolymer binders in combination with straw fiber reinforcement. Additionally, a strength prediction methodology based on a GA-BP neural network is proposed. A GA-BP neural network prediction model is constructed, with the levels of the three biopolymers serving as the input layer and the uniaxial compressive strength as the output layer, integrating the optimization capabilities of the genetic algorithm. Meanwhile, support vector machine (SVM) was also introduced as an alternative predictive model in order to facilitate the performance comparison of models and select the optimal solution. The model is subjected to training, validation, and predictive generalization analyses to verify its effectiveness and robustness. Furthermore, microscopic characterization techniques are employed to elucidate the interaction mechanisms at the interface between the biopolymer binder, fibers, and soil particles, providing insights into the physical reinforcement effects of the fibers and the adsorption phenomena of polar molecules. The overarching aim of this research is to offer a theoretical foundation for civil engineering construction in the Loess Plateau region.

## 2. Materials and Methods

### 2.1. Materials

**Figure 1:**
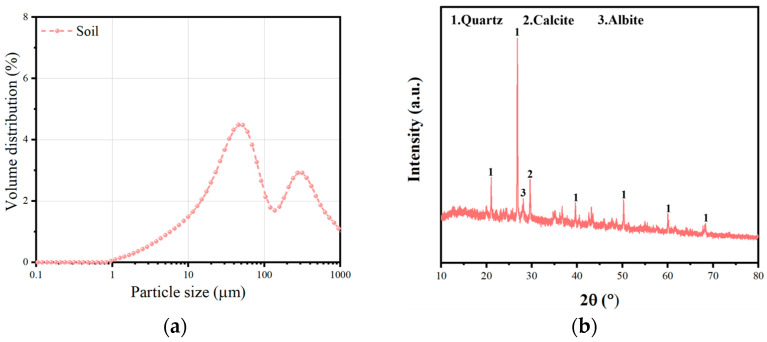
Particle size distribution curve (**a**) and XRD diffraction pattern (**b**) of the soil.

The loess employed in this study was sourced from Yulin City, Shaanxi Province. Its particle size distribution curve and mineralogical composition are depicted in Figure 1. The particle size distribution curve indicates that the particle volume distribution presents a unimodal pattern in relation to particle size. A peak is observed at a particle diameter of approximately 20–30 μm, suggesting that particles around this size account for the largest proportion in the soil. It can be inferred that the soil is mainly composed of medium-sized particles, with coarse and fine particles being relatively scarce. Additionally, the XRD spectrum reveals the presence of minerals such as quartz, calcite, dolomite, and clinochlore in the soil. The high intensity of the quartz diffraction peak demonstrates that it is the predominant mineral component. The relatively prominent peaks of calcite and dolomite also indicate their significant presence. Detailed chemical composition and technical properties are presented in Table 1 and Table 2, respectively. The maximum dry density and optimal moisture content of the soil were determined to be 1.861 g/cm^3^ and 13.58%, respectively.

**Figure 2:**
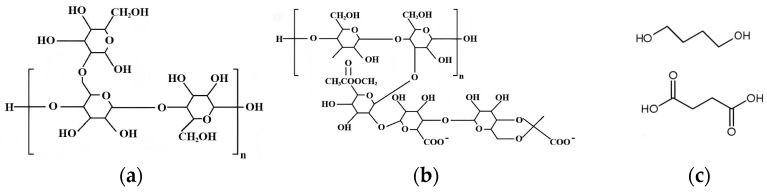
Chemical structures of (**a**) GG; (**b**) XG; and (**c**) PBS.

The XG employed in this study was procured from Shanghai Maikelin Biochemical Technology Co., Ltd. (Shanghai, China), while the GG was obtained from Hefei Qian-sheng Biotechnology Co., Ltd. (Hefei, China). GG is a polysaccharide extracted from the seeds of the guar plant, scientifically known as *Cyamopsis tetragonoloba* [27]. It consists of D-galactose and D-mannose units in a ratio of 1:2 [28]. The chemical structures of XG and GG are shown in Figure 2. The chemical and physical properties of the two biopolymers are presented in Table 3.

Typically produced by xanthomnas campestris, XG is a polysaccharide that is generated through the fermentation process of a carbohydrate-based substrate, such as glucose or sugars, derived from sources like cornstarch. Its backbone consists of a linear chain of D-glucose units linked by 1,4-glycosidic bonds, with a charged trisaccharide side chain attached to every two glucose units. The resulting side chains are formed from D-glucuronic acid elements connected to two D-mannose units [29].

Polybutylene Succinate (PBS) exhibits different fibrous or lamellar morphologies due to its thermoplastic aliphatic polyester properties [30]. PBS was provided by PTT-MCC Biochem (Bangkok, Thailand), with a melt flow rate (MFR) value of 22 g/10 min (at 190 °C under a load of 2.16 kg), a melting temperature of 115 °C, a glass transition temperature of −36 °C, and a density of 1.26 g/cm^3^.

### 2.2. Experimental Design

The specimen preparation process was divided into three key stages: soil pretreatment, biopolymer hydration, and mixing and molding. Initially, the soil was dried at 105 °C for 24 h, then crushed and sieved through a 2 mm mesh. The soil was evenly spread within a sealed container, and deionized water was added to achieve the target moisture content, as determined by compaction tests. The container was then sealed and left undisturbed for 24 h to ensure uniform moisture distribution. The mix proportions for specimen molding are shown in Table 4. Initially, the polymer powders were pre-blended with deionized water in a 1:10 ratio and subjected to agitation at 200 rpm for 15 min to facilitate gel formation. Subsequently, the biopolymer gels were hermetically sealed and stored for 24 h to enable complete hydration and water absorption reactions. Finally, the hydrated soil was combined with the gel and mixed in a forced mixer for 15 min. The mixture was subsequently molded into cylindrical specimens with dimensions of Φ50 mm × H50 mm through static compression molding.

### 2.3. Test Methods

#### 2.3.1. Mechanical Properties Test

Unconfined compressive strength tests were conducted on specimens of biopolymer–fiber composite-stabilized soil using a compression testing machine. The loading rate was set at 1 mm/min. The dimensions of the specimens were Φ50 mm in diameter and H50 mm in height. Each group comprised six samples, and the arithmetic mean of the strengths of these six samples was adopted as the final test result for the respective group.

#### 2.3.2. Micro Properties Tests

To investigate the formation mechanism of the strength in biomacromolecule–fiber composite-stabilized soil structures and to elucidate their microscopic structural characteristics, the powder samples were analyzed using a Smartlab SE X-ray diffraction (XRD) diffractometer. The scan rate was configured at 10° per minute, with the scanning angular range set from 10° to 80°. Fourier-transform infrared (FT-IR) spectroscopy was performed on the samples using a Nicolet iS5 spectrometer, with 32 scans conducted per sample. The spectral range was from 400 cm^−1^ to 4000 cm^−1^, with a resolution of 4.00 cm^−1^. Thermogravimetric-differential scanning calorimetry (TG-DTG) analysis was carried out using a TA-SDT650 thermogravimetric analyzer, with the temperature range set from 25 °C to 800 °C. The samples were heated at a rate of 10 °C per minute, and nitrogen was employed as the protective gas to prevent carbonization. Additionally, the microstructural morphology of the samples was examined utilizing a Zeiss Sigma 300 scanning electron microscope (SEM).

### 2.4. Analysis Methods

The backpropagation (BP) neural network, as a supervised learning multilayer feedforward network trained via the error backpropagation algorithm, has been extensively employed across numerous research domains [31,32]. It effectively models the complex nonlinear relationships between inputs and outputs through the collaborative integration of the input layer, hidden layer, and output layer. During the development of the BP neural network model for multi-component polymer-stabilized soil’s mechanical properties, challenges such as the complexity of experimental combinations, data limitations, and network architecture selection have been encountered.

This study aims to investigate the impact and predictive modeling of composite formulation ratios within a multi-polymer system on the mechanical properties of fiber-reinforced stabilized soils, thereby maintaining a constant fiber dosage as the baseline control parameter. Among the numerous influencing factors, the type of polymer has been recognized as a decisive determinant of soil stabilization performance. Consequently, in the construction of the model, it has been essential to treat the polymer type as a pivotal input variable. Based on engineering practices, the maximum unconfined compressive strength of the biopolymer-stabilized soil specimens has been selected as the indicator for evaluating mechanical performance, which has been employed as the output data in the output layer.

Genetic algorithm (GA), an optimization technique that models biological evolution processes to seek optimal solutions, has been chosen as the optimization approach owing to its unique mechanism of simulating biological evolution. GA is particularly suited for complex tasks such as performance prediction in multivariate systems, as it can effectively handle multiple influencing factors simultaneously and has broad exploration capabilities within the solution space [33].

Support vector machine (SVM), a statistical learning-based algorithm, excels in regression and classification across disciplines [34,35]. By maximizing the margin between data points and the decision boundary, it efficiently handles high-dimensional, nonlinear, and small-sample data. Using kernel functions, SVM projects inputs into higher-dimensional spaces to model complex relationships robustly. For polymer-stabilized soil mechanics, SVM provides a parameter-efficient alternative to neural networks, improving generalization with limited data. Thus, it serves as a complementary tool for geotechnical predictions, enhancing accuracy and model stability.

Based on this premise, three predictive models—BP neural network, GA-BP neural network, and SVM—have been constructed, and their performance in forecasting the mechanical properties of multi-component biopolymer-stabilized soils has been evaluated and compared. The conventional BP neural network has been designated as the baseline model, while the network parameters of the GA-BP model have been fine-tuned by leveraging genetic algorithms, aiming at enhancing prediction accuracy and stability. The SVM model, with kernel functions being utilized, has been presented as an alternative approach by which high-dimensional, nonlinear data can be effectively handled with fewer parameters and potentially improved generalization can be achieved, especially when dealing with limited datasets. A comparative analysis of these three models has been carried out, providing valuable insights into their respective strengths and limitations and enabling the most suitable modeling technique to be selected for complex geotechnical performance prediction in multi-polymer stabilization systems [36].

#### 2.4.1. Determine the Coding Method

Based on the mechanism whereby XG and GG form a three-dimensional network through hydrogen bonding and electrostatic interactions to encapsulate soil particles, along with the reinforcement effects of PBS interpenetrating and filling the structure, it is hypothesized that different combinations of biopolymers can significantly enhance the soil’s compressive strength. Therefore, uniaxial compressive strength is selected as the core mechanical property indicator, as it directly reflects the soil’s capacity to resist axial pressure under unconfined conditions and intuitively demonstrates the internal structural changes induced by biopolymer stabilization. Additionally, this indicator is characterized by a simple testing method and reliable data, which facilitates the use of the GA-BP model for the prediction and optimization of mechanical properties. Therefore, the model employed key factors influencing the mechanical properties of polymer-stabilized soils—namely, the dosages of GG, XG, and PBS—as input variables, with the 7-day compressive strength serving as the output.

**Figure 3:**
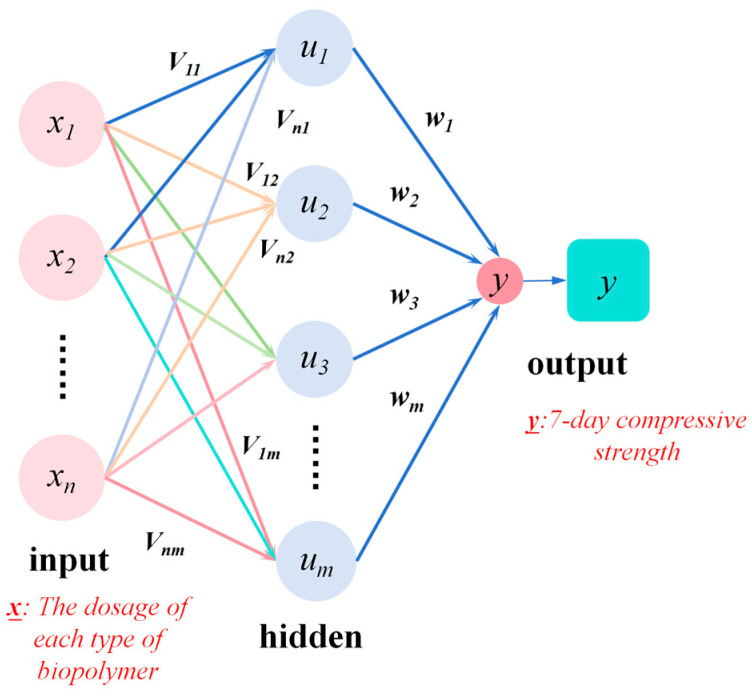
BP neural network structure.

Through the adjustment of the network’s connection weights, the performance prediction was achieved (Figure 3). In the BP neural network architecture, the input information was weighted and propagated to the hidden layer, followed by further transmission through weighted connections to the output layer. The multiple nodes within the hidden layer conducted complex nonlinear computations, generating the model’s output, which was then compared against the experimental data to determine the error. During the training process, the chain rule was employed to compute the partial derivatives of the error with respect to each neuron and connection weight layer-by-layer [37]. The cycle of “forward propagation–backpropagation–forward propagation” was repeated until the error met the predefined criteria. Furthermore, the SVM model was developed concurrently as a comparison in order to validate the universality of the models. This model is characterized by its ability to map input data into a high-dimensional feature space through kernel functions. Its advantages in handling small-sample nonlinear problems are highlighted, and its predictive results are used to perform complementary validation with the GA-BP model.

#### 2.4.2. Generation of the Initial Population

Once the encoding method was determined, the initial population was generated based on the network topology to establish the number of link weights. All connection weights were encoded using a real-valued encoding approach to construct the population, with the population size set to “gen_num”. For each individual chromosome, the impact of varying polymer dosage compositions was evaluated through prediction calculations using the BP network, and the resulting computational errors relative to the measured data were employed as the fitness value. The fitness function, denoted as “fitness”, is expressed in Equation (1):

(1) $$\begin{array}{l} A_{1}=\tan sig(W_{1}*X+b_{1})\\ \hat{y}=W_{2}*A_{1}+b_{2}\\ fitness={\left(\hat{y}-y\right)}^{2} \end{array}$$

where “*X*” represents the input data, *ŷ* denotes the model-predicted output, “*W*_1_” corresponds to the weight matrix connecting the input layer and the hidden layer, “*b*_1_” is the bias vector associated with the input-to-hidden connections, “*W*_2_” relates to the weight matrix connecting the hidden layer and the output layer, and “*b*_2_” is the bias vector associated with the hidden-to-output connections.

#### 2.4.3. Selection, Crossover, and Mutation

During each evolutionary cycle, new populations were generated through selection, crossover, and mutation. Individuals with better fitness, based on training error, were retained, while poorer ones were discarded. High-quality individuals underwent genetic operations to produce improved offspring. The process was repeated until a predefined fitness level was achieved.

In the GA-BP model, connection weights were encoded as chromosomes using real-valued coding. Optimization through genetic operations improved the population, and the best chromosomes were decoded into network parameters for prediction. This approach enabled the BP neural network to efficiently model the nonlinear interactions among multiple biopolymers and predict their effects on soil stability.

## 3. Predictive Model Construction

### 3.1. Data Selection and Normalization

The BP neural network was employed for the construction, training, and simulation prediction using MATLAB (R2025a)’s Neural Network Toolbox. The Randperm function was initially used to generate randomly permuted indices, which were then applied to shuffle the rows of the dataset. This process facilitated data randomization, effectively eliminating data continuity and ensuring that the model had access to randomly sampled data for training and testing. Consequently, bias was minimized, and the robustness of the model was enhanced. A total of 46 datasets obtained from uniaxial compression tests were used. Of these, 80% of the data were allocated as the training set, while the remaining 20% served as the testing set to validate the model’s accuracy. The input variables were constrained within the range [0, 2.5]. Additionally, data normalization was performed to improve the network’s efficiency, simplify computation, and enhance prediction accuracy, as demonstrated by the transformation function in Equation (2).

(2) $$x^{*}=\frac{x-x_{\min}}{x_{\max}-x_{\min}}$$

Here, the maximum value of the dataset is denoted by *x_max_*, while the minimum value is represented by *x_min_*. In this study, the sample data were subjected to preprocessing for normalization through the utilization of the mapminmax function in MATLAB(R2025a). Subsequently, upon the completion of model training, the actual predicted values were derived by performing inverse normalization on the processed data.

In the code implementation, several key settings were employed to prevent overfitting. In the study, 80% of the dataset was strictly allocated as the training set and 20% as the testing set. During model training, an early stopping mechanism was implemented via a callback function, which monitored the validation loss and halted training if no improvement was observed for a specified number of epochs (10). Additionally, L2 regularization was integrated into the loss function to penalize large parameter values, thereby controlling model complexity. These measures were applied using built-in modules from the deep learning framework, ensuring robustness and generalization despite the limited dataset size.

### 3.2. Setting the Optimization Parameters

A single-hidden-layer BP neural network architecture was constructed using the newff function, with the trainlm function employed for network training. To determine an appropriate number of hidden-layer nodes, a trial-and-error approach was adopted: the network was initially trained with a relatively small number of hidden-layer nodes, which were then incrementally increased (up to a threshold). Ultimately, the number of hidden-layer nodes that minimized the network error was selected. Specific network testing parameters can be found in Table 5. The parameter selection for the BP neural network model was guided by the need to balance model complexity, convergence speed, and prediction accuracy. The maximum number of iterations was set to 1000 to allow sufficient training cycles for convergence without excessive computation time. A convergence error threshold of 1 × 10^−6^ was chosen to ensure high precision in model training, preventing overfitting and underfitting. The learning rate of 0.005 was selected to optimize the training process by providing a stable yet efficient update step. Connection weights between input, hidden, and output layers were initially assigned with a threshold of 15 for the hidden layer and 10 for the output layer to facilitate effective learning progress. The parameters were tuned through iterative experimentation and validation, leading to optimized values that enhanced the model’s predictive performance and stability, ensuring reliable convergence during training.

Furthermore, the parameter configuration was carried out with great care in the process of constructing the standalone SVM model, as demonstrated in the code. As shown in Table 6, the radial basis function (RBF) kernel was selected and specified by the option -t 2. The penalty parameter C was set to 4.0, which serves to strike a delicate balance between prediction errors (in the regression context) and model complexity. The RBF kernel parameter gamma was fixed at 0.7, playing a crucial role in regulating the influence scope of individual training samples. Given that the task at hand was regression analysis, the task type -s was designated as 3. Additionally, the epsilon value of the insensitivity loss function was established at 0.01 to define the allowable error margin between the predicted and actual values. These parameter settings were meticulously determined to empower the model to effectively capture the intricate nonlinear interactions among multiple biopolymers and accurately forecast their impacts on soil stability.

### 3.3. Genetic Algorithm Implementation

**Figure 4:**
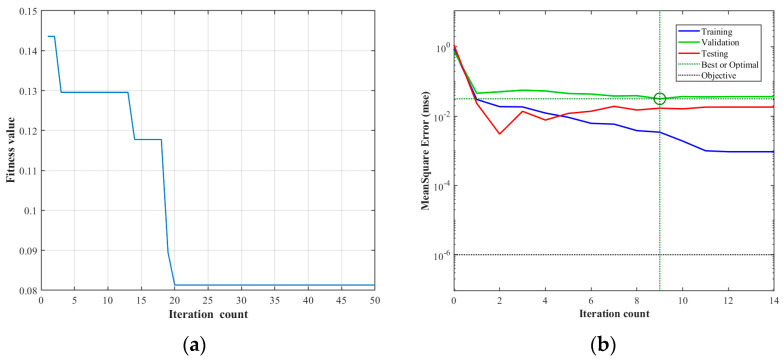
Convergence diagram of network training. (**a**) Adaptation curve, (**b**) MSE iteration curve.

To minimize the residuals between predicted values and expected values during prediction using the BP network, an error matrix paradigm between the predicted values and the expected values of the prediction samples was selected as the output of the objective function. The fitness function employed a ranking-based fitness assignment function, expressed as fitness = ranking (obj), where obj represents the output of the objective function. The parameter settings for the genetic algorithm utilized in this study are listed in Table 7. The created GA-BP neural network was trained based on the previously configured network parameters. Through the analysis of the adaptation curve changes depicted in Figure 4a, it was observed that the adaptation reached its minimum value of 0.08144 at the 20th iteration.

## 4. Analysis and Discussion

### 4.1. Mechanical Properties

**Figure 5:**
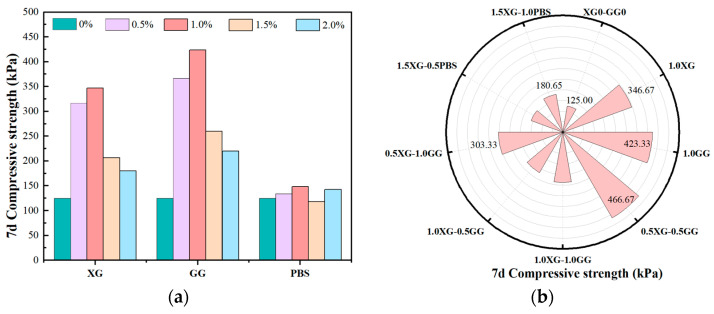
7d compressive strength of biopolymer–fiber-reinforced composite-stabilized soil. (**a**) single-doping group, (**b**) multiple-doping group.

The UCS test results for biopolymer–fiber-reinforced composite-stabilized soil are presented in Figure 5. Figure 5a illustrates the influence of the independent incorporation of XG, GG, and PBS on the strength characteristics of soil specimens. Comparison with the control group shows that single additives, such as GG and XG, markedly improve strength; it is observed that the most pronounced enhancements in strength are induced by GG and XG, which are attributed to their ability to form cohesive films on the surface of soil particles. Specifically, the 7-day strength of the specimen containing 1.0% GG is measured at 423.33 kPa, representing an increase of 239% relative to the control group at 125 kPa, and surpassing the 346.67 kPa observed in the 1.0% XG specimen. The single-additive groups provide a baseline that confirms GG and XG as the most effective in strengthening soils, while PBS alone shows limited improvement, highlighting the importance of biopolymer type.

The improvement in strength is primarily attributed to the formation of a polymer film that encapsulates soil particles, thereby inducing aggregation effects [38]. The cross-linking of polymeric molecular chains contributes to the enhancement of soil stability by reinforcing the bonding between particles, leading to increased cohesion and crack resistance. The increase in compressive strength is, thus, a direct consequence of these modifications in the soil microstructure [39]. It is noteworthy that the effect of biopolymer content on strength is characterized by an initial increase, followed by a decline at higher concentrations. When the content exceeds an optimal range, a state of oversaturation within the soil matrix may be reached, resulting in the formation of weak interface layers due to excess polymers. This phenomenon is associated with reduced bonding strength and the generation of micro-voids at the interfaces. Furthermore, an overabundance of polymer chains can lead to internal stress concentration, which promotes microcrack initiation and propagation, ultimately diminishing the overall strength [40]. The optimal biopolymer content is therefore estimated to be within the range of 0.5% to 1.0%.

Figure 5b presents the effects of combined usage of XG, GG, and PBS on soil compressive strength. The specimen containing 0.5% XG and 0.5% GG demonstrates the highest strength of 466.67 kPa, which equals a 273% increase over the control group, meeting the specified standards for the sub-base layers of secondary and lower-grade highways. The strengths observed for specimens with 0.5% XG–1.0% GG and 1.0% XG–0.5% GG are 303.33 kPa and 220 kPa, respectively, indicating a notable decline compared to the 0.5% XG and GG combination. The enhanced strength resulting from the co-application of 0.5% XG and GG is ascribed to the synergistic effects of the two biopolymers, which promote the formation of cross-linked networks on soil particle surfaces [41]. Molecular chains are capable of forming ionic bridges and hydrogen bonds with clay particles via carboxyl (-COOH) and hydroxyl (-OH) groups, which are present on the molecular structures [42]. During co-crosslinking, the hydroxyl groups on GG and the carboxyl groups on XG facilitate additional hydrogen bonding, further strengthening molecular interactions. These interactions lead to the development of a densely interconnected network structure, thereby significantly enhancing the soil particle bonding strength and reducing microcrack pathways [43,44]. The addition of fibers as reinforcement elements is also beneficial, as it inhibits crack development and improves tensile strength and may interact with polymers to further reinforce the structural integrity of the stabilized soil. The combined effects of polymeric and fibrous reinforcements are therefore responsible for the observed improvements in strength and overall stability [45].

However, the multicomponent nature of these polymer systems introduces considerable complexity and variability, as subtle changes in material proportions can significantly affect strength. This complexity hampers precise control and systematic optimization in practical applications. Relying solely on traditional experimental methods is inefficient for identifying optimal formulations among numerous multi-component configurations, since this approach is resource-intensive and limits the scope of exploration. Therefore, a strength prediction approach based on a GA-BP neural network model has been developed, enabling efficient and accurate optimization of polymer compositions and facilitating the advancement of high-performance biopolymer soil reinforcement systems.

### 4.2. Comparative Analysis of BP, GA-BP Neural Networks, and SVM Model for Predicting Mechanical Properties

**Figure 6:**
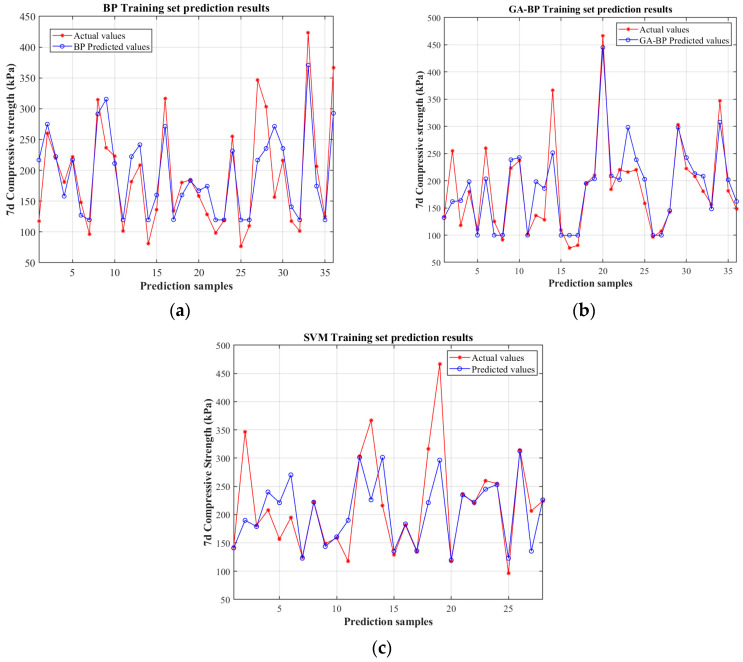
Comparative analysis of predicted vs. actual values on the training set for the BP prediction model (**a**), GA-BP model (**b**), and SVM model (**c**).

A comprehensive demonstration of the predictive performance exhibited by the traditional BP neural network, the genetic algorithm (GA)-optimized BP neural network, and the support vector machine (SVM) is illustrated by Figure 6. A comparative analysis between the predicted and actual compressive strength values within the training samples is depicted through these figures. Consistent input parameters are employed for the configuration of all models, and appropriate optimization techniques are utilized for their training—the Levenberg–Marquardt algorithm being applied to neural networks and kernel function optimization to SVM. Following the completion of the training process, reverse normalization is performed on the outputs to enable a direct comparison with empirical data.

The predictive performance of the traditional BP neural network and the GA-BP neural network is comprehensively illustrated in Figure 6a,b. Both models were configured with identical network parameters—including the number of neurons in the hidden layer, the maximum iteration count, the learning rate, and the termination criterion—and were trained utilizing the Levenberg–Marquardt algorithm. Subsequent to the training process, the outputs were subjected to reverse normalization to facilitate a direct comparison with the empirical data. The plots reveal that the GA-BP model yields predictions that are substantially closer to the actual measurements, exemplified by the minimal deviations and higher consistency throughout the dataset, particularly in the higher strength regimes. Conversely, the predictions derived from the conventional BP neural network exhibit more pronounced discrepancies, indicating a relatively weaker capacity for capturing the complex nonlinear relationships inherent in the data [46]. This superior performance of the GA-BP model can be ascribed to the evolutionary optimization of initial weights and thresholds, which effectively enhances the convergence behavior and mitigates the issues related to local minima entrapment. As shown in Figure 6c, the predictive performance of the SVM model is found to be noticeably inferior to that of the GA-BP model. Significant deviations between the predicted values and the actual data are observed, along with poor consistency and increased dispersion, especially in the high-strength regions. This could be due to suboptimal kernel parameter choices or the inherent difficulty in capturing the complex nonlinear relationships in this specific dataset with the current SVM configuration. When dealing with such complex data, the selection of kernel functions and the relatively limited parameter settings of SVM are unable to precisely match the intrinsic structure of the data. Consequently, the intricate relationships within the data cannot be effectively captured, and the predictive accuracy is thus constrained.

**Figure 7:**
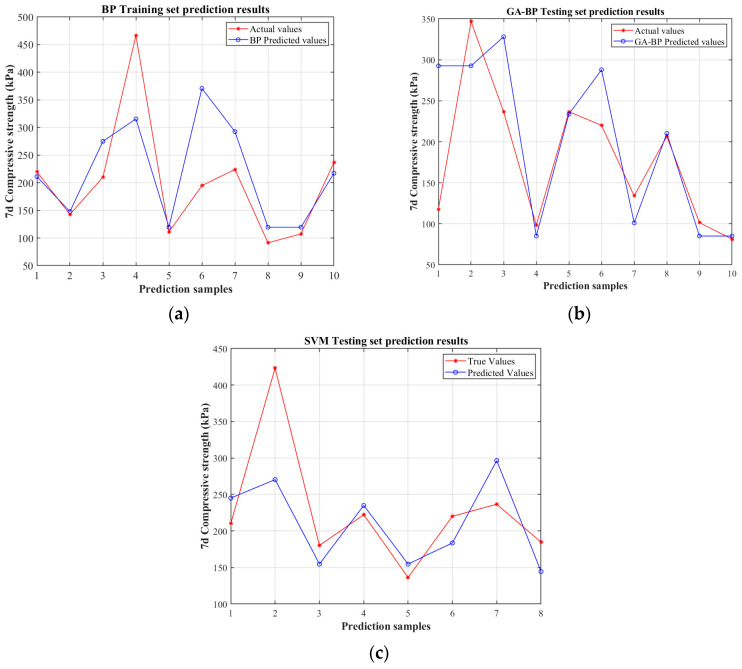
Comparative analysis of predicted vs. actual values on the testing set for the BP prediction model (**a**), GA-BP model (**b**), and SVM model (**c**).

The comparison between the predicted and actual strength values for the test set, as illustrated in Figure 7a,b, demonstrates that the ten predicted strength values derived from the GA-BP model exhibit a significantly closer alignment with the empirical measurements, whereas the predictions obtained from the conventional BP neural network display comparatively larger discrepancies relative to the experimental data. An observable trend indicates that the overall prediction accuracy improves progressively with an increasing number of test samples, approaching an asymptotic convergence. This phenomenon underscores the enhanced generalization capability and predictive efficacy of the GA-optimized BP neural network, relative to the standard BP regression model. Such performance enhancement can be attributed to the intrinsic advantage conferred by the integration of the genetic algorithm, which operates through iterative optimization processes to refine the initial weights and thresholds of the network. This optimization effectively mitigates the risk of entrapment in local minima, thereby facilitating a more robust exploration of the solution space and substantially augmenting the model’s capacity for accurate extrapolation. Consequently, the results substantiate that the evolutionary algorithm integration significantly elevates the predictive competency, ensuring more reliable assessments in complex geotechnical applications. In contrast, the predictions obtained from the SVM model, as shown in Figure 7c, tend to be less accurate relative to the actual values. The predicted strength values exhibit larger discrepancies and scatter, particularly in some of the higher strength samples, indicating that the SVM’s predictive performance in this context is comparatively limited. This is mainly attributed to the complex characteristics of the prediction data in this case, which may include high noise levels or unique distributions. SVM depends on kernel functions to map data into a high-dimensional space for achieving linear separability. Nevertheless, while the SVM’s performance in this instance appears less effective, its inclusion provides a comprehensive perspective when assessing the robustness of different modeling approaches for geotechnical performance prediction.

### 4.3. Predictive Indicator Analysis

**Figure 8:**
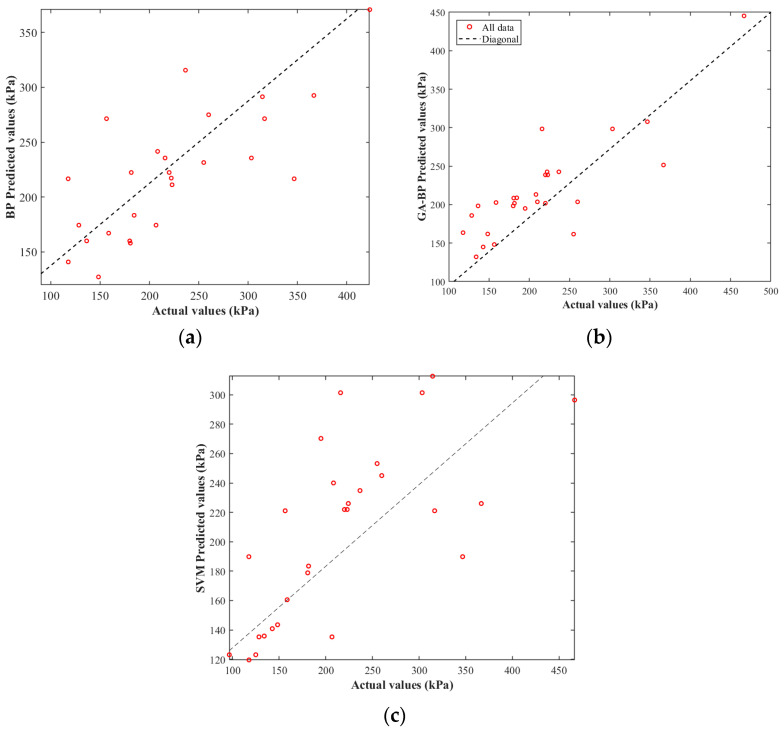
Regression analysis plot of predicted vs. actual values for BP Model (**a**), GA-BP Model (**b**), and SVM Model (**c**).

Figure 8a,b illustrate the regression plots depicting the relationship between the actual and predicted compressive strength values generated by the BP neural network and the GA-BP neural network, respectively. The horizontal axis represents the true measured values, while the vertical axis corresponds to the predicted outputs. These plots serve to visually assess the predictive performance and the degree of correlation between the model outputs and empirical data. In the case of the BP neural network, the predicted values exhibit a discernible tendency to deviate from the ideal 45-degree line, indicating that while the model captures the overall trend, systematic errors and residual discrepancies are present. Conversely, the regression plot associated with the GA-BP model demonstrates a markedly stronger correlation, with the data points more closely aligning along the diagonal reference line. This signifies that the GA-BP model facilitates a more accurate approximation of the true strength values, with reduced residual deviations. The enhanced alignment observed in the GA-BP regression plot underscores its superior predictive accuracy and generalization capability, attributable to the genetic algorithm’s optimization of initial weights and thresholds, which effectively mitigates local minima entrapment endemic to conventional neural networks. Consequently, the regression analysis exemplifies the significant advancement achieved through the integration of evolutionary algorithms, leading to improved model robustness and estimation precision in the context of compressive strength prediction.

As shown in Figure 8c, the regression plot of the SVM model exhibits a more dispersed pattern, with many data points significantly deviating from the ideal diagonal line. Although some data points align with the trend, the overall correlation is weaker compared to neural network models. The scattered distribution of data points indicates that, under the current parameter settings, the SVM has limited capability to accurately predict compressive strength, particularly in the high-strength range. The relatively loose clustering of data points near the diagonal line suggests that the SVM’s generalization ability is somewhat constrained within this specific dataset, a characteristic that highlights its continued predictive limitations compared to the GA-BP model.

**Figure 9:**
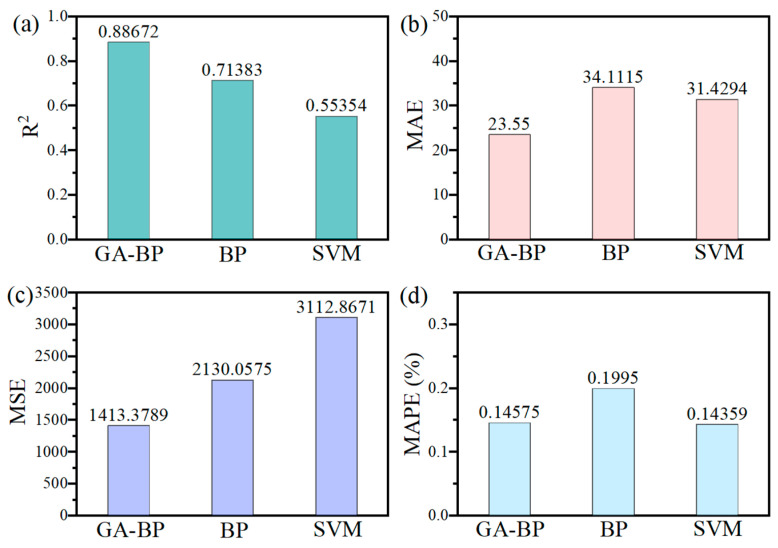
(**a**) R^2^, (**b**) MAE, (**c**) MSE, and (**d**) MAPE for three types of prediction models.

The comprehensive quantitative basis for comparing the three algorithmic models is provided by the four sets of performance evaluation metrics presented in Figure 9, namely the coefficient of determination (R^2^), mean squared error (MSE), mean absolute error (MAE), and mean absolute percentage error (MAPE). R^2^ is calculated to quantify the proportion of variance in the dependent variable that has been captured by the model, with higher values indicating that a greater degree of explanatory power has been achieved. MSE is determined by averaging the squared differences between predicted and actual values, through which larger errors are penalized more heavily. MAE is obtained by computing the average absolute difference, offering a direct measure of prediction accuracy where large errors are not overemphasized. MAPE is derived by expressing the average absolute error as a percentage of the actual values, enabling comparisons to be made across different scales or units. The formulas for each evaluation index are shown in (3) to (6).

(3) $$R^{2}=1-\frac{\sum_{i=1}^{n}{\left(y_{i}-{\hat{y}}_{i}\right)}^{2}}{\sum_{i=1}^{n}{\left(y_{i}-\bar{y_{i}}\right)}^{2}}$$

(4) $$MSE=\frac{1}{n}\sum_{i=1}^{n}{\left(y_{i}-{\hat{y}}_{i}\right)}^{2}$$

(5) $$MAE=\frac{1}{n}\sum_{i=1}^{n}\left|y_{i}-{\hat{y}}_{i}\right|$$

(6) $$MAPE=\frac{1}{n}\sum_{i=1}^{n}\left|\frac{A_{i}-F_{i}}{A_{i}}\right|$$

where n is the number of samples, y_i_ is the actual value, $\bar{y}$ denotes the mean of the actual observations, and ${\hat{y}}_{i}$ is the predicted value of the model. A_i_ denotes the i-th actual observed value (true value), while F_i_ represents the i-th predicted value.

The predictive efficacy of the GA-BP model, in comparison with the conventional BP neural network and the SVM model, is comprehensively evaluated through the performance comparison presented in Figure 9, with four key statistical metrics—R^2^, MAE, MSE, and MAPE—being employed. In Figure 9a, the coefficient of determination (R^2^) is illustrated. An R^2^ value of 0.88672 is achieved by the GA-BP model, indicating that an excellent fit has been attained and suggesting that approximately 88.7% of the variance in the observed data is captured. In contrast, a lower R^2^ of 0.71383 is exhibited by the BP model, reflecting that diminished explanatory power has been demonstrated. An R^2^ of 0.55354 is attained by the SVM model, demonstrating that a moderate but notably weaker correlation has been established compared to the neural network approaches.

Regarding predictive accuracy, the MAE values presented in Figure 9b reveal that a mean absolute error of 23.55 is achieved by the GA-BP model. This signifies that minimal average deviations have been observed between its predictions and actual values, thus indicating that high precision has been attained. An MAE of 34.11 is possessed by the BP model, implying that larger average prediction errors have been incurred. The MAE of the SVM model is approximately 31.43, which is relatively close to that of the GA-BP but slightly higher, suggesting that less precise predictions have been made by the SVM compared to the GA-BP, yet they are comparable to those of the BP model.

The evaluation of MSE, depicted in Figure 9c, further affirms the performance differences. A considerably lower MSE of 1413.3789 is recorded by the GA-BP network compared to 2130.0575 for the BP model. The MSE of the SVM is around 3112.8671, which is higher than those of both the GA-BP and BP models. Since larger deviations are penalized more heavily by MSE, its reduction in the GA-BP model indicates that heightened stability has been achieved and that the occurrence of significant prediction errors has been reduced.

Figure 9d presents the MAPE metric. A mean percentage error of 0.14575 is exhibited by the GA-BP model, notably lower than the 0.1995 observed in the BP model. The MAPE of the SVM is approximately 0.14359, very close to that of the GA-BP, indicating that comparable predictive performances have been delivered by the SVM and GA-BP in terms of percentage errors. This similarity may be attributed to the SVM’s ability to handle nonlinear relationships efficiently, which, with appropriate kernel parameters, can achieve predictive accuracy comparable to the optimized neural network model.

Overall, the collective analysis of these four metrics decisively indicates that the best overall predictive performance among the three models is exhibited by the genetic algorithm-enhanced BP neural network. The highest model fit (R^2^) and the lowest error metrics (MAE, MSE, and MAPE) are demonstrated by the GA-BP model, showcasing the beneficial impact of genetic algorithm-based optimization in enhancing initial weight configurations and convergence behavior. Although not outperforming the GA-BP in most metrics, competitive results are shown by the SVM model, especially in MAPE, suggesting that it is a viable alternative for strength prediction given proper parameter tuning. Compared to the other two models, relatively weaker modeling capabilities are exhibited by the traditional BP neural network, although it is capable. The improvements in model fit (R^2^) and error metrics (MAE, MSE, and MAPE) exemplify the beneficial impact of genetic algorithm-based optimization, which effectively enhances initial weight configurations and convergence behavior, resulting in more accurate, stable, and reliable strength predictions across the dataset.

### 4.4. Microscopic Mechanism Analysis

**Figure 10:**
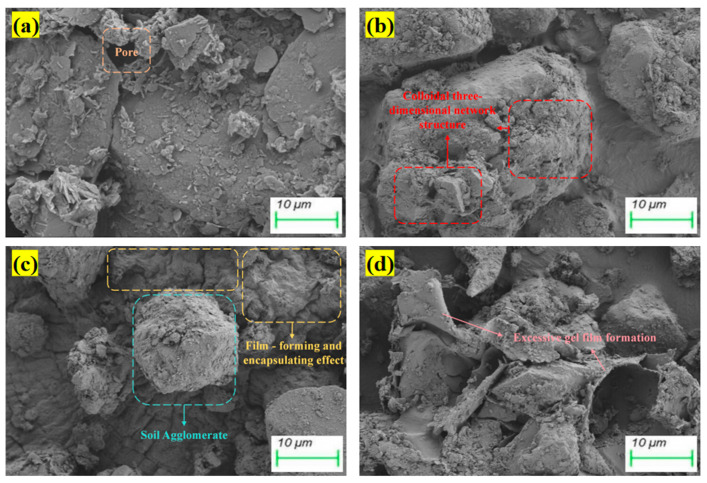
SEM images of biopolymer–fiber-reinforced composite-stabilized soil: (**a**) Control group; (**b**) 0.5GG; (**c**) 0.5XG-0.5GG; (**d**) 1.0XG-1.0GG.

The analysis of the micro-morphological characteristics of the biopolymer–fiber-reinforced composite stabilized soil is conducted to unveil the composite stabilization mechanism of biopolymers and fibers. As shown in Figure 10a, the soil was constituted by soil particles, including silica phases, some layered mineral fragments, and other fine particles, which formed a certain stabilized structure through straw fiber reinforcement. However, large pores and inter-structural gaps were distinctly observed within the framework. The incorporation of biopolymers was found to cause clay particles to be encapsulated within a thin, uniform film coating structure, thereby providing adhesion (Figure 10b–d). Although a partially formed network-like biopolymer structure was observed in the 0.5GG group, which contributed to the binding and stabilization of soil particles, its coverage effect was limited. The relatively short molecular structure of GG, characterized by its strong hydrogen bonding capacity, was capable of rapidly encapsulating soil particles during hydration, forming a uniform film that effectively enhanced adhesion between particles and facilitated localized hardening [47].

It is noteworthy that, within the 0.5XG-0.5GG experimental group, extensive regions of effective three-dimensional network crosslinking and membrane coverage were observed (Figure 10c). The widespread presence of these hardened membrane areas served as evidence of beneficial synergistic interactions among the molecular structures. The longer molecular chains of XG demonstrated a greater capacity for interchain crosslinking, which enabled the formation of more durable and stable three-dimensional networks. Simultaneously, the shorter chains of GG exhibited rapid hydration and film-forming abilities, which facilitated the early-stage formation of membrane structures. The two polymers could further increase the spatial crosslinking density of the network through interchain hydrogen bonds and electrostatic interactions, thereby promoting a substantial increase in the volume of the polymer-crosslinked network within the system [48]. This synergistic mechanism enhanced the soil’s cohesiveness and structural stiffness, leading to a notable improvement in its mechanical properties [49]. When combined with the toughening effect of straw fiber reinforcement, the three-dimensional network stabilization system was further strengthened, resulting in a significant enhancement in the soil sample’s strength characteristics, consistent with the observed mechanical performance outcomes.

From Figure 10d, it can be observed that the 1.0XG-1.0GG group exhibited prominent curled or wrinkled structures, which may be attributed to an excessive amount of biopolymer within the system. An overabundance of biopolymers results in an overly viscous system environment, where interactions among polymer molecules become more complex and intense. The originally more ordered molecular arrangements and interaction patterns are disrupted, and increased entanglement and interweaving between molecular chains and straw fibers occur. This makes it difficult to form uniform and stable structures. During drying or curing, the excessive entanglement of these molecules, coupled with uneven contraction, leads to localized stress concentrations, subsequently causing curling and deformation of the structure [50]. Therefore, precise control over the optimal dosage of biopolymers within the straw fiber-reinforced system is essential.

## 5. Conclusions

Aiming to address the limitations posed by the complex multi-component ratios in biopolymer-stabilized soils within multivariate systems, which hinder rapid and accurate performance prediction, a GA-optimized BP neural network model was developed. The strength prediction and performance evaluation of biopolymer-stabilized soils in multivariate systems were conducted using both the traditional BP model and the enhanced GA-BP model. The following conclusions were drawn:

(1) The optimal dosage range of biopolymers for fiber-reinforced composite stabilized soil is approximately 0.5–1.0%. The combined doping of 0.5XG-0.5GG achieves the highest compressive strength of 466.67 kPa, a 273% enhancement, meeting the sub-base intensity criterion for secondary and lower-grade highways.
(2) Compared to the traditional BP neural network and SVM, the GA-BP model—with an R^2^ of 0.8867, an MAE of 23.55, an MSE of 1413.38, and a MAPE of 0.14575—demonstrates significantly improved accuracy and stability. The integration of genetic algorithm optimization for initial weights and convergence has enhanced the model’s predictive performance, outperforming BP by approximately 30–34% in errors and showing better results than SVM. This confirms that GA-BP is the most reliable and precise approach for predicting the uniaxial compressive strength.
(3) The strength mechanism of biopolymer-stabilized soils is attributed to the further enhancement of the spatial crosslinking density within the polymer three-dimensional network, achieved through interchain hydrogen bonding and electrostatic interactions in the reinforced skeletal framework based on straw fibers. Soil stabilization is realized through the formation of a film-like hardened coating that encapsulates soil particles.

## Figures and Tables

**Table 1:** Chemical composition of the materials (wt.%).

Material	CaO	SiO_2_	Al_2_O_3_	Fe_2_O_3_	SO_3_	MgO	K_2_O	TiO_2_	Other
Soil	12.472	60.315	15.219	5.382	0.07	2.478	3.033	0.787	0.244

**Table 2:** Basic physical properties of soil.

Material	Natural Moisture Content (%)	Plastic Limit (%)	Liquid Limit (%)	Plasticity Index (I_p_)	Maximum Dry Density (g/cm^3^)	Optimum Water Content (w_op_)
Soil	10.12	7.22	24.57	17.35	1.861	13.58%

**Table 3:** Chemical and physical properties of biopolymers.

Material	Molecular Mass (g/mol)	Viscosity (mPa.s)	pH	Grain Size	Form
XG	268.5	1680	7.8	80 mesh	Powder
GG	522.6	5368	6.2	100 mesh	Powder

**Table 4:** Mix proportion for specimen molding.

Sample	XG (%)	GG (%)	PBS (%)	Straw Fiber (%)	Sample	XG (%)	GG (%)	PBS (%)	Straw Fiber (%)
XG-GG-0	0	0	0	0.4%	PBS-1.0	0	0	1.0	0.4%
XG-0.5	0.5	0	0		PBS-1.5	0	0	1.5	
XG-1.0	1.0	0	0		PBS-2.0	0	0	2.0	
XG-1.5	1.5	0	0		0.5XG-0.5GG	0.5	0.5	0	
XG-2.0	2.0	0	0		1.0XG-1.0GG	1.0	1.0	0	
GG-0.5	0	0.5	0		0.5XG-1.0GG	0.5	1.0	0	
GG-1.0	6	1.0	0		1.0GG-0.5XG	1.0	0.5	0	
GG-1.5	0	1.5	0		1.5XG-0.5PBS	0	1.5	0.5	
GG-2.0	0	2.0	0		1.5XG-1.0PBS	0	1.5	1.0	
PBS-0.5	0	0	0.5		-				

**Table 5:** BP network model setting parameters.

Model Parameters	Maximum Number of Iterations	Convergence Error	Learning Rate	Connection Weights Between Input Layer and Hidden Layer	Hidden Layer Threshold	Connection Weights Between Hidden Layer and Output Layer	Output Layer Threshold
Value	1000	1 × 10^−6^	0.005	15	10	10	5

**Table 6:** SVM model setting parameters.

Model Parameters	Kernel Type (-t)	Penalty Parameter (C)	RBF Kernel Parameter (Gamma)	Task Type (-s)	Epsilon Value of Insensitivity Loss Function
Value	2 (RBF kernel)	4.0	0.7	3	0.01

**Table 7:** Operating parameters of the genetic algorithm.

Population Size	Genetic Generations	Selection Function Parameters	Crossover Function Parameters	Mutation Function Parameters
80	10	0.08	2	3

## Data Availability

All data are contained within the article.
